# Tranexamic Acid to Treat Life-threatening Hemorrhage in Prostate Cancer Associated Disseminated Intravascular Coagulation with Excessive Fibrinolysis

**DOI:** 10.7759/cureus.428

**Published:** 2015-12-22

**Authors:** Oksana Prokopchuk-Gauk, Kelsey Brose

**Affiliations:** 1 Department of Laboratory Medicine and Pathology, University of Alberta; 2 Department of Medicine, University of Saskatchewan, Royal University Hospital

**Keywords:** prostate neoplasm, tranexamic acid, lysine analogue, disseminated intravascular coagulation, primary fibrinolysis, hemorrhage, hypofibrinogenemia

## Abstract

This case illustrates the acute onset of life-threatening bleeding in a 57-year-old male with treatment-resistant metastatic prostate cancer. Laboratory results confirmed the presence of disseminated intravascular coagulation (DIC). Despite aggressive transfusion support, his consumptive coagulopathy and persistent bleeding could not be controlled, and his need for blood products began to outpace the available supply. Our patient had declined further chemotherapy treatment for his underlying aggressive prostate cancer and would accept only palliative care.

Both thrombosis and bleeding are known to coexist during DIC. In our patient, his hemorrhagic clinical condition and laboratory results supported the presence of DIC with an excessive fibrinolytic process in the setting of metastatic prostate cancer. Following careful consideration of potential risks and benefits, the decision was made to administer the antifibrinolytic agent tranexamic acid to control bleeding. Initiation of this treatment led to rapid bleeding cessation without thrombotic complication. Although controversial, this treatment was life-saving in our patient and allowed hospital discharge. He remained transfusion independent for his remaining four weeks of life following discharge, and ultimately died at home of multi-organ failure related to his cancer.

## Introduction

The presentation of a patient with life-threatening hemorrhage in association with disseminated intravascular coagulation (DIC) can be a frightening experience. Identification of the presence of DIC is paramount; for early determination of its etiology and immediate intervention can be lifesaving.

## Case presentation

A 57-year-old male receiving palliative care for treatment-resistant metastatic prostate cancer was admitted to his local, primary-care hospital for management of worsening bone pain and acute renal insufficiency. His medical profile also included hypertension, non-insulin dependent diabetes, and dyslipidemia. Shortly after admission, the patient developed a sudden onset of massive epistaxis and frank hematuria. Laboratory investigations demonstrated an acutely elevated international normalized ratio (INR) measuring > 8.0 and progressing anemia in the absence of anticoagulant medications. Despite administration of two 10 mg doses of vitamin K and transfusion with fresh frozen plasma and packed red blood cells, bleeding could not be controlled. Temporary hemodynamic stability was achieved with fluid support, and he was urgently transferred to our tertiary care center.

Upon arrival, the patient was tachycardic and tachypneic with progressing respiratory compromise. His blood pressure was stable, and he was afebrile. He appeared markedly pale with prominent multiple petechiae, ecchymoses, and hematomas visible on his trunk, abdomen, and extremities. Nasal packing became blood-soaked. His gums were visibly bleeding, and intravenous sites were oozing. Cardiovascular examination revealed normal heart sounds and a flat, jugular venous pressure. Diffuse, coarse rhonchi were audible throughout both lung fields. The abdomen was soft with active bowel sounds present.

**Figure 1:**
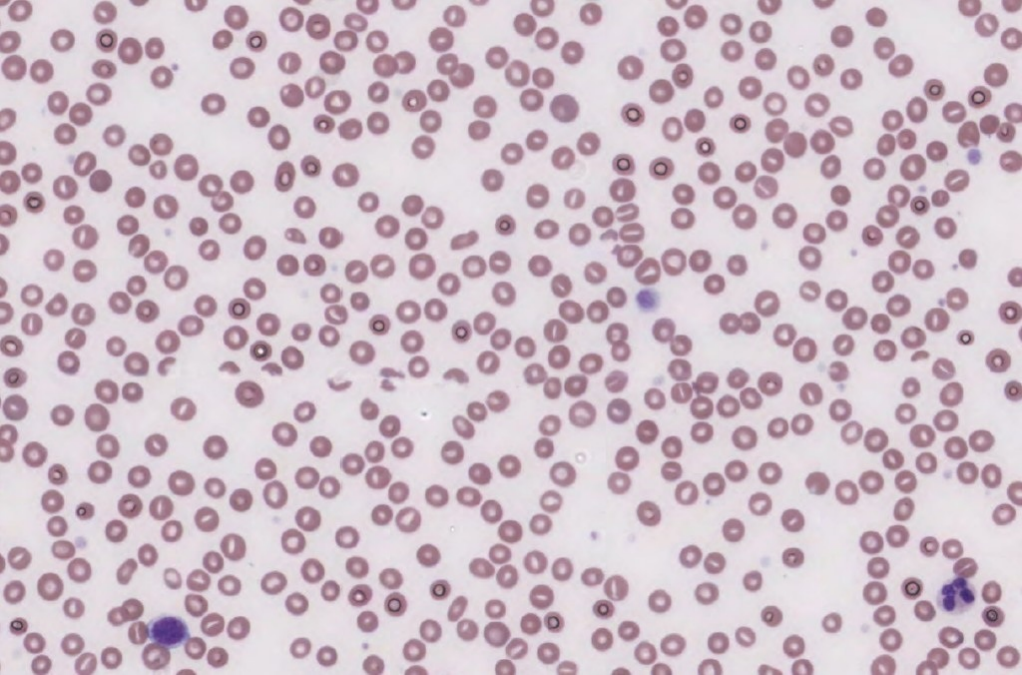
Blood smear of a 57 year old man with a bleeding diathesis, showing rare schistocytes and thrombocytopenia.

The blood picture showed a hemoglobin of 81 g/L (normal 135-175), a platelet count of 84 x 10^9^/L (normal 150-400), and a leukocyte count of 13.5 x 10^9^/L (normal 4.0-11.0) with an elevated neutrophil count. Creatinine and urea were elevated at 115 μmol/L (normal 45-125) and 20 mmol/L (normal 3.7-7.0), respectively. Transaminases were normal; bilirubin was elevated at 28 μmol/L (normal 2-22) and lactate dehydrogenase at 380 U/L (normal 100-190). Coagulation parameters included an elevated, post-vitamin K INR of 2.6 (normal 0.8-1.2) and partial thromboplastin time (PTT) of 43 seconds (normal 26-36). The D-dimer was markedly elevated at 5479 μg/L (normal 0-500) with an undetectable fibrinogen of <0.5 g/L (normal 1.50-4.00). Urinalysis was positive for hemoglobin with more than 100 red blood cells present per high power field, with no nitrites or white blood cells present. A chest x-ray showed mild basal lung atelectasis with no focal consolidation. A Doppler ultrasound of the legs showed no clot in the popliteal or femoral veins. The patient’s peripheral blood smear is depicted in Figure 1.

A clinical diagnosis of DIC was made, presumed secondary to the presence of metastatic prostate cancer as no other precipitant of this bleeding diathesis could be identified. Supportive transfusions with blood products were initiated immediately. Nasal packing soaked with a 5% tranexamic acid solution was applied. During his first seven days of admission, he received intensive transfusion therapy, including 18 units of packed red blood cells, 41 units of frozen plasma, 20 adult doses of platelets, and 130 units (13 adult doses) of cryoprecipitate. Only temporary improvements achieved in red cell, platelet, and coagulation parameters. Blood cultures were confirmed to be negative after five days of incubation. Clinical monitoring was continually negative for evidence of thrombotic complication. Unrelenting epistaxis was his greatest source of blood loss.

Unfortunately, his consumptive coagulopathy and persistent bleeding could not be controlled with transfusion support only, and his need for blood products began to outpace the available supply. He declined further chemotherapy treatment for his prostate cancer, maintaining his wish to receive palliative care with a goal of returning home.

As a final measure to control bleeding in this transfusion resistant bleeding diathesis, we proposed administration of the systemic antifibrinolytic agent tranexamic acid to our patient. Following discussion, including the potential thrombotic risk with this medication in the setting of malignancy and DIC, he agreed to receive tranexamic acid with the hope of gaining control of his bleeding. Therefore, tranexamic acid was initiated at a dose of 1500 mg orally every six hours.

Remarkably, his bleeding stopped completely within hours of his first tranexamic acid dose. His clinical condition dramatically improved, and he became transfusion independent within 36 hours. He was discharged home shortly thereafter and subsequently continued to take tranexamic acid four times daily. He was once again offered chemotherapy treatment for his prostate cancer, and he declined. He remained at home throughout the next several weeks, enjoying his final days surrounded by family and friends. There was no recurrence of bleeding or evidence of acute arterial or venous thrombosis. Four weeks following hospital discharge, the patient died peacefully at home. No autopsy was performed, with the cause of death attributed to multisystem organ failure secondary to his metastatic cancer.  

## Discussion

DIC is a syndrome characterized by clinical manifestations of both clotting and bleeding. Clotting occurs when activation of intravascular clotting factors leads to fibrin deposition, while bleeding is a result of the coagulation factor and platelet consumption [1] and activation of intrinsic anticoagulant mechanisms [2].

Under normal circumstances, a balance is maintained between systemic prothrombotic and anticoagulant pathways. Thrombin concurrently activates endogenous procoagulant and anticoagulant mechanisms of fibrin deposition and degradation, respectively. The process of thrombin-mediated fibrin degradation is known as secondary fibrinolysis* *[3], whereby thrombin stimulates the release of intrinsic plasminogen activators from endothelial cells, including tissue plasminogen activator (t-PA) and urokinase plasminogen activator (u-PA) to generate plasmin [2]. Plasmin is then responsible for degradation of both the precursor fibrinogen to produce fibrinogen degradation products (fibrinogenolysis), and for cleavage of fibrin into fibrin split products (fibrinolysis).  When the delicate, hemostatic balance is tipped in favor of bleeding as a result of excessive secondary fibrinolysis, this process is known as DIC [4].

There are many well-recognized precipitants of DIC, including malignancy, sepsis, and severe trauma [5]. The incidence of acute DIC in the setting of cancer is estimated to be 7-20% [1]. In most malignancy-associated cases of DIC, it is typically the thrombotic component that predominates [4]. The mainstay of DIC management is the treatment of the underlying cause [1, 5].

Clinical manifestations of DIC include the development of venous, microcirculatory and, uncommonly, arterial thrombosis as a result of excess intravascular fibrin deposition. Bleeding is frequently seen at arterial or venous puncture sites or areas of a skin incision or trauma. Spontaneous bleeding may also occur from mucocutaneous tissues, appearing as petechiae, ecchymoses, and hematomas [5].

Laboratory blood test results may be helpful in the diagnosis of DIC. Coagulation parameters, including the PTT and INR, are prolonged due to consumption of coagulation factors, with accompanying anemia and thrombocytopenia. Fibrin degradation products and fibrin split products, including the D-dimer, are found to be increased in the serum while fibrinogen is low given its consumption. The peripheral blood smear will have numerous schistocytes, which are a consequence of red blood cell fragmentation by fibrin deposited in the microcirculation, and confirm a low platelet count. 

Primary (hyper) fibrinolysis is a pathological mechanism of direct plasminogen activation and fibrinogen degradation, independent of coagulation and fibrin formation, which leads to profound bleeding [3, 6]. Primary fibrinolysis has been known to exist without DIC as a unique entity and is responsible for causing a hemorrhagic state in the setting of metastatic prostate cancer and acute promyelocytic leukemia [2-3]. The clinical manifestations of primary fibrinolysis include marked mucocutaneous bleeding without micro or macrovascular thrombotic complications.

On blood analysis in primary fibrinolysis (which is technically the only fibrinogenolysis), fibrinogen is low, and fibrinogen degradation products are in excess; however, there is no increase in fibrin split products, and the D-dimer is within normal limits [7]. Schistocytes are not usually present in the blood smear as red cell fragmentation does not occur in the absence of fibrin deposition. Platelets are normal as there is no increase in their consumption as a result of thrombus formation. Coagulation times are typically prolonged as a consequence of plasmin-induced factor proteolysis and fibrinogen degradation product interference with fibrin polymerization. A comparison of laboratory results in DIC and primary fibrinolysis are listed in Table 1.

**Table 1:** Comparison of the features of DIC and primary fibrinolysis DIC = disseminated intravascular coagulation, PTT = partial thromboplastin time; INR = international normalized ratio

Lab Parameter	DIC	Primary Fibrinolysis
Platelets	Low	Normal
Schistocytes	Present	Absent/Rare
D-dimer	High	Normal
Fibrinogen	Low	Low
PTT	Prolonged	Prolonged
INR	Prolonged	Prolonged

It is possible for both primary and secondary fibrinolysis to coexist, with a single underlying disease process [5, 7]. In this setting, a hemorrhagic clinical presentation will predominate, and coagulation parameters will reflect pathologic processes of both fibrinolytic process. DIC with excessive fibrinolysis is a recognized, rare complication of prostate cancer and is associated with a poor prognosis. A recent, retrospective case series of 42 patients identified that with chemotherapy, the median survival in this patient population was 26 weeks, and included complete reversal of coagulopathy in 20% of patients with metastatic disease [8]. Without additional chemotherapy, median survival was found to be only four weeks. Regardless of treatment, none of the patients in this series developed thrombotic complications.

In the setting of metastatic prostate cancer, the mechanism of primary hyperfibrinolysis appears to be related to the expression of u-PA by prostate cancer cells [6]. It is thought that u-PA functions to promote tumor invasion via proteolysis of surrounding tissues with plasmin. The excess u-PA within the systemic circulation, therefore, allows for activation of plasminogen to plasmin, independent of fibrin formation. The activated plasmin acts primarily on the fibrin precursor fibrinogen, producing excessive fibrinogen degradation products, but no significant amount of fibrin split products.

In our patient, the combination of a diffuse bleeding diathesis and admission bloodwork results including thrombocytopenia, severe hypofibrinogemia, high D-dimer, and prolonged PTT and INR, were convincing for the presence of DIC. However, the low schistocyte count in the peripheral blood smear and dramatic bleeding diathesis of the patient led us to suspect the presence of a concurrent, primary hyperfibrinolytic process, particularly in the setting of his aggressive metastatic prostate cancer. He showed no signs of thrombotic complications in the setting of his hemorrhagic coagulopathy.

Management of DIC is particularly challenging when the underlying cause cannot be acutely addressed. Current pharmacologic, supportive-care strategies are controversial. Intravenous heparin has been suggested to attenuate bleeding in DIC by reducing consumption of clotting factors and the secondary fibrinolytic process, in addition to inhibiting activation of coagulation to reduce thrombotic complications [1, 5]. The use of antifibrinolytic agents to treat hemorrhage in the context DIC has been considered a relative contraindication by many, as it is thought that these medications may potentiate clot formation due to inhibition of the secondary fibrinolytic process [2].

Tranexamic acid and ε-aminocaproic acid are well-established, antifibrinolytic agents. As lysine analogues, they function by preventing plasmin binding to fibrinogen or fibrin, thereby preventing proteolysis by plasmin [9]. Tranexamic acid may be administered orally, has a longer half-life, and has a significantly greater potency of effect in comparison to ε-aminocaproic acid. Case reports of fatal thrombosis following use of lysine analogues in DIC have been published in the literature [10]. In contrast, there is more recent evidence of successful tranexamic acid and ε-aminocaproic acid use to treat bleeding complications associated with excessive fibrinolysis in acute promyelocytic leukemia [5] and metastatic prostate cancer [3, 7].

We hypothesized that bleeding in our patient persisted due to the predominance of a primary fibrinolytic process with DIC, likely secondary to his aggressive metastatic prostate cancer. Due to the concern of further hemorrhagic complications with heparin administration, we chose to use an antifibrinolytic agent to arrest bleeding.  Not only did bleeding stop shortly following tranexamic acid administration, but we were able to safely discharge him home for the remainder of his palliation according to his wishes. His short, four-week survival was expected and is consistent with recently published survival data [8]. The outcome of his clinical course ultimately supported our hypothesis.

## Conclusions

In conclusion, we suggest that use of an antifibrinolytic agent be considered in the setting of transfusion-resistant bleeding when clinical and laboratory evidence supports the presence of excessive fibrinolysis. We recognize that this practice is controversial, but there may exist outcome benefits that outweigh the potential risks of using antifibrinolytic medications to treat life-threatening hemorrhage accompanying DIC in the setting of malignancy.

## References

[1] Levi M (2009). Disseminated intravascular coagulation in cancer patients. Best Pract Res Cl Ha.

[2] Colman RW, Rubin RN (1990). Disseminated intravascular coagulation due to malignancy. Semin Oncol.

[3] Hodson A, Hunt BJ (2007). A bleeding tongue – acute presentation of fibrinolysis. Int J Lab Hematol.

[4] Francis JL (1989). Haemostasis and Cancer. Med Lab Sci.

[5] Rocha E, Paramo JA, Montes R, Panizo C (1998). Acute generalized, widespread bleeding. Diagnosis and management. Haematologica.

[6] Kohli M, Kaushal V, Mehta P (2003). Role of coagulation and fibrinolytic system in prostate cancer. Semin Thromb Hemost.

[7] Cooper DL, Sandler AB, Wilson LD, Duffy TP (1992). Disseminated intravascular coagulation and excessive fibrinolysis in a patient with metastatic prostate cancer: response to epsilon-aminocaproic acid. Cancer.

[8] Hyman DM, Soff GA, Kampel LJ (2011). Disseminated intravascular coagulation with excessive fibrinolysis in prostate cancer: a case series and review of the literature. Oncology.

[9] Royston D (1995). Blood sparing drugs: aprotinin, tranexamic acid and ε-aminocaproic acid. Int Anesthesiol Clin.

[10] Ratnoff OD (1969). Epsilon aminocaproic acid – a dangerous weapon. New Engl J Med.

